# Morphological description of *Pintomyia* (*Pifanomyia*) *veintemillasi* n. sp., a new sand fly species from the sub-Andean region of Bolivia

**DOI:** 10.1186/s13071-022-05433-1

**Published:** 2022-09-19

**Authors:** Eddy Martinez, Renato Leon, Andrei Daniel Mihalca, Jean-Pierre Dujardin, François Le Pont

**Affiliations:** 1 Instituto de Investigación en Salud y Desarrollo, IINSAD; Cátedra de Parasitología, Facultad de Medicina, Universidad Mayor de San Andrés, UMSA, La Paz, Bolivia; 2 Laboratorio de Entomología Médica & Medicina Tropical LEMMT, Colegio de Ciencias Biológicas y Ambientales, COCIBA, Universidad San Francisco de Quito, Cumbayá, 150157 Quito, Ecuador; 3 Department of Parasitology and Parasitic Diseases, University of Agricultural Sciences and Veterinary Medicine of Cluj-Napoca, Cluj-Napoca, Romania; 4 Institut de Recherche Pour Le Développement, IRD, Montpellier, France; 5 93100 Montreuil, France

**Keywords:** Sand flies, Taxonomy, Evansi series, Cryptic species, Andean foothills

## Abstract

**Background:**

Most sand fly species are located in the Americas; some act as vectors of leishmaniasis and other human diseases. In Bolivia, about 25% of Neotropical species have been identified, and only a few have been implicated as vectors of cutaneous and visceral leishmaniasis. A new species of anthropophilic sand fly from the sub-Andean region of Alto Beni is described herein.

**Methods:**

A large systematic entomological survey was carried out in a subtropical humid forest located in the Marimonos mountain range, at around 900 m altitude, in the municipality of Palos Blancos, Sud Yungas Province, Department of La Paz, Bolivia. Sand flies were captured over a period of 26 months between January 1982 and February 1984, at the ground and canopy level, using both CDC light traps and protected human bait. A total of 24,730 sand flies were collected on the ground, distributed in 16 species, and 3259 in the canopy, with eight species. One of these species was labeled as *Pintomia* (*Pifanomyia*) *nevesi*, although certain morphological features allowed us to doubt that it was that taxon. To define the identity of this sand fly, a re-evaluation (this work) was recently carried out through morphological analyses and measurements of the available specimens mounted on Euparal, previously labeled as *Pi*. (*Pif*.) *nevesi*.

**Results:**

Based on the morphological traits and measurements, the re-evaluated specimens were definitively identified as a new sand fly species, *Pintomyia* (*Pifanomyia*) *veintemillasi*, closely related to *Pi*. (*Pif*.) *nevesi* and *Pintomyia* (*Pifanomyia*) *maranonensis* within the Evansi series. This new sand fly was the third most numerous anthropophilic species at the floor (6.2%) and the second most numerous anthropophilic at the canopy (35.1%).

**Conclusions:**

A new anthropophilic sand fly species is described as *Pi*. (*Pif*.) *veintemillasi* n. sp. This sand fly species was caught at about 900 m altitude in the Marimonos mountain range, a highly endemic area for cutaneous and mucosal leishmaniasis due to *Leishmania* (*Viannia*) *braziliensis*. Therefore, this species could be involved in the leishmaniasis transmission in the sub-Andean foothills of Alto Beni, Department of La Paz, Bolivia.

**Graphical Abstract:**

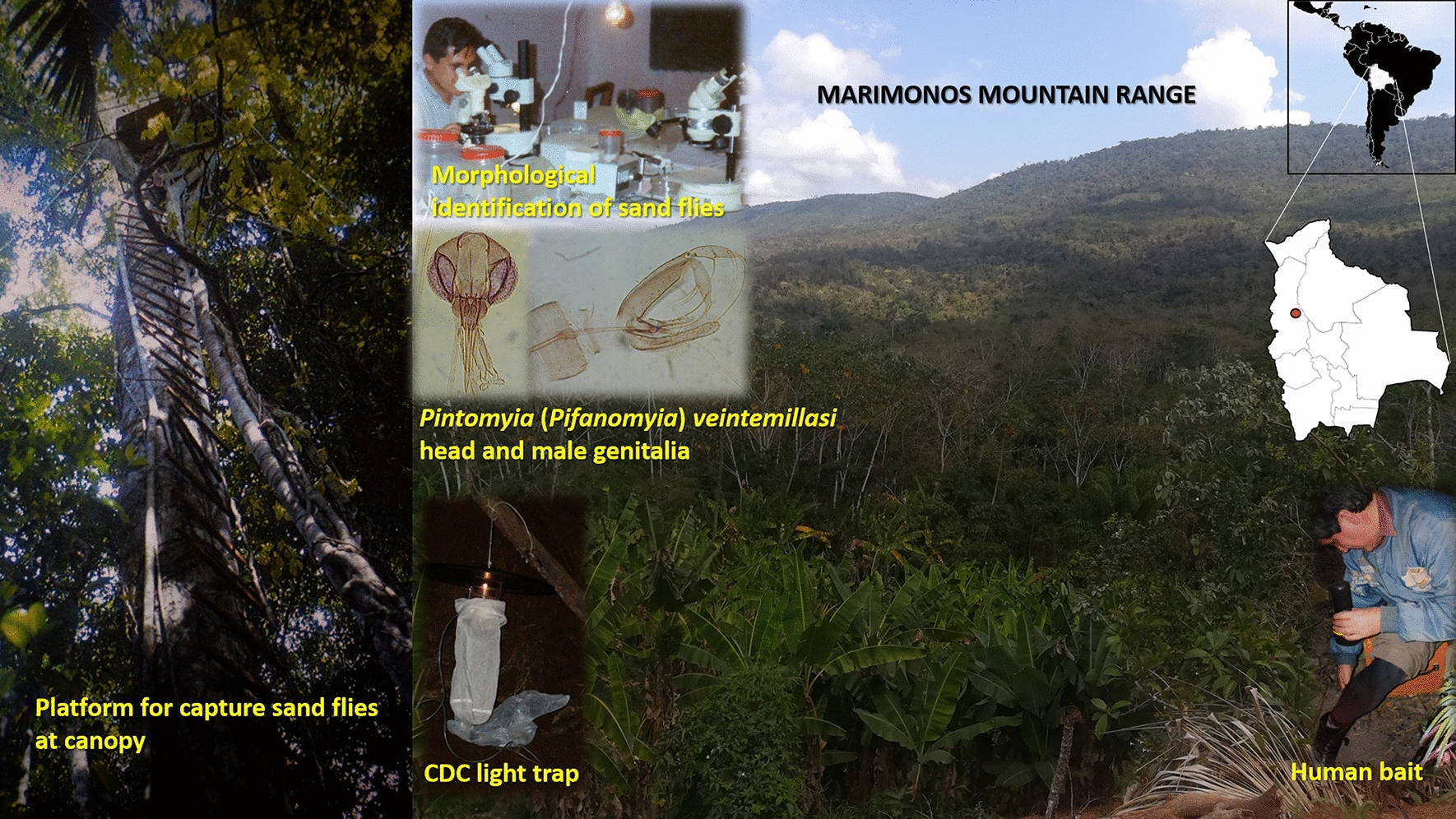

## Background

Phlebotomine sand flies are natural vectors of around 30 kinetoplastids of the genus *Leishmania*, the bacterium *Bartonella bacilliformis*, and some arboviruses. The medical importance of sand flies is mostly related to their ability to transmit to humans at least 20 well-known *Leishmania* species, causing different clinical forms of leishmaniasis, with around two million people affected worldwide every year [1, 2].

For Neotropical sand flies, in the 1990s, Duncan and Young published what was considered the most complete compilation of the American phlebotomine sand flies at the time [1, 3]. Later, Galati proposed a new classification based on phylogenetic analysis which elevated to genus most of the subgenera proposed in the earliest classification [1, 2] and is currently the most widely accepted approach. According to Galati, the current list of American sand flies includes 539 species/subspecies (522 current and 17 fossil) distributed in 23 genera and 20 subgenera, three groups of species, and 30 series of species [2]. The taxonomic groups and series proposed are frequently used by sand fly taxonomists in the New World, although they are not familiar in the Old World and thus are not always recognized. Around 135 sand fly species have been identified in Bolivia (≈25% of Neotropical species), distributed in 21 genera, 14 subgenera, 17 series of species, and one group of species [2–4].

The genus *Pintomyia* Costa Lima, 1932 includes the subgenus *Pintomyia* Ortiz & Scorza, 1963 with eight species, and *Pifanomyia* Ortiz & Scorza, 1963 with 72 species. Most of the species of the subgenus *Pifanomyia* are distributed in the Andean region [2]. Within this subgenus, *Pintomyia* (*Pifanomyia*) *nuneztovari anglesi* Le Pont & Desjeux, 1984 [syn. *Pintomyia* (*Pifanomyia*) *nuneztovari* Ortiz, 1954] has been implicated in the transmission of zoonotic leishmaniasis in domestic environments in the sub-Andean area of Bolivia, as the proven vector of *Leishmania amazonensis* and a suspected vector of *Leishmania braziliensis* [1, 2]. This subgenus has been divided into seven series of sand fly species. Among them, the Evansi series Galati, 2003 comprises four species including *Pintomyia* (*Pifanomyia*) *nevesi* Damasceno & Arouck, 1956 [2], which is widely present in South America and is distributed from the foothills of the Andes mountains of Colombia, Ecuador, Peru, and Bolivia to the lowlands in the five Brazilian states of Para, Acre, Rondonia, Maranhao, and Mato Grosso [2]. In the past, this species was included in the Verrucarum group [3].

*Pintomyia* (*Pif.*) *nevesi* is a sylvatic species, originally described in 1956 from two male specimens collected in a station on the Capim River in the state of Para, Brazil [5]. Unfortunately, in the original description, some measurements were imprecise, making the morphological identification of specimens difficult and raising doubts as to their true identity.

Similarly, a few decades ago a female from a collection of 12 specimens from the sub-Andean foothills of La Paz, Bolivia, was identified as *Pi.* (*Pif.*) *nevesi* [6]. But lacking information on the males that were collected, and given the distance and difference in altitude compared with the type locality of *Pi.* (*Pif.*) *nevesi* in Brazil, the report from Bolivia raised controversy.

Later, Dujardin and Le Pont [7] examined the intraspecific variation in the traditional morphometric traits used for the identification of sand flies. The comparison of metric characteristics was carried out from 12 species from Bolivia between conspecific populations belonging to the same eco-region and between conspecific populations from different eco-regions. Unfortunately, when comparing the populations of *Pi.* (*Pif.*) *nevesi* from the sub-Andean and Amazonian regions, anomalies forced these specimens to be excluded from this study.

Afterwards, a molecular study was carried out by Beati et al. [8] to identify seven sand fly species from the Verrucarum group (sensu Young & Duncan, 1994) from Peru, where two females and one male *Pi.* (*Pif.*) *nevesi* from the sub-Andean region of San Martin, were included. However, the female specimens were eliminated from the study, due to significantly different sequences in comparison to clearly identified male *Pi.* (*Pif.*) *nevesi*.

Considering the dubious identity of sub-Andean *Pi.* (*Pif.*) *nevesi* from Bolivia and Peru, the identity of past Bolivian sand fly collections identified as *Pi*. (*Pif*.)* nevesi* was thoroughly revised. Based on the identification key of the species of the Evansi series [2], we provide both male and female descriptions of a new species close to *Pi*. (*Pif*.)* nevesi* and the taxonomic characteristics of the related species. Recently, the closely related species *Pi.* (*Pif.*) *maranonensis* [15] was identified naturally infected by *Leishmania* (*Viannia*) *peruviana* in the Eastern Andes of northern Peru [9], increasing the interest and importance of further studies of the Evansi series.

## Methods

A large systematic entomological survey was conducted between January 1982 and February 1984 in a subtropical humid forest at the municipality of Palos Blancos, Sud Yungas Province, Department of La Paz, Bolivia (Alto Beni region). Phlebotomine sand flies were collected monthly for 26 months, at both the ground and canopy levels using Centers for Disease Control and Prevention (CDC) light traps and protected human bait. The latter procedure was privileged, considering the importance of the anthropophilic species as potential vectors of leishmaniasis. As expected, the captures using protected human bait were mainly females. As additional information, the male/female sex ratio collected with CDC light traps over 1 year in human environments was 0.58 for wood-wall houses and 0.77 for adobe-wall houses.

A total of 24,730 phlebotomine sand flies comprising 16 species were collected at the floor, and 3259 sand flies comprising eight species at the canopy. At the floor, *Pi.* (*Pif.*) *veintemillasi* represented 6.2% (1526 specimens) of anthropophilic species, ranking third only after *Psychodopygus carrerai carrerai* Barreto, 1946 (the proven vector of *L. braziliensis*) and *Psychodopygus hirsutus hirsutus* Mangabeira, 1942, without a known vectorial role in the transmission of leishmaniasis. At the canopy, *Pi.* (*Pif.*) *veintemillasi* represented the second most common anthropophilic species, with 35.1% (1027 specimens) of the captures, exceeded only by *Pintomyia* (*Pifanomyia*) *serrana* Damasceno & Arouck, 1949 [10]. The current description of *Pi.* (*Pif.*) *veintemillasi* as a new species is possible using the only available mounted biological material (collected in February 1982, using both capture procedures) of this important anthropophilic sand fly, which was previously misidentified as *Pi.* (*Pif.*) *nevesi*.

Both male and female individuals of the species of interest were initially sorted based on the similarity of the pleura pigmentation. Sand fly specimens were sorted using a dissecting microscope and placed in 70% ethanol. For species identification, sand flies were treated using the Abonnenc technique [11] and mounted in Euparal media. Ten to 12 specimens of each sex and its related species were used for the morphometric analysis. The measurements are given in millimeters (*a* = average). The classification, character nomenclature, genus, and subgenus name abbreviations follow Galati [2] and Galati et al. [12].

Although many specimens of this new species have been captured in the past, we only have available those from the collection, which serve for the present description. The others, misidentified as *Pi*. (*Pif*.) *nevesi*, were unfortunately discarded. However, we will program new captures for further studies including the similar species of the Evansi series, using molecular tools and geometric morphometry.

## Results

From morphological analysis and measurements of mounted specimens, previously labeled as *Pi*. (*Pif*.) *nevesi*, a new sand fly species was identified.

**Family Psychodidae Newman, 1834**

**Genus** ***Pintomyia*** **Costa Lima, 1932**

**Subgenus (** ***Pifanomyia*** **) Ortiz & Scorza, 1963**

**Fig. 1:**
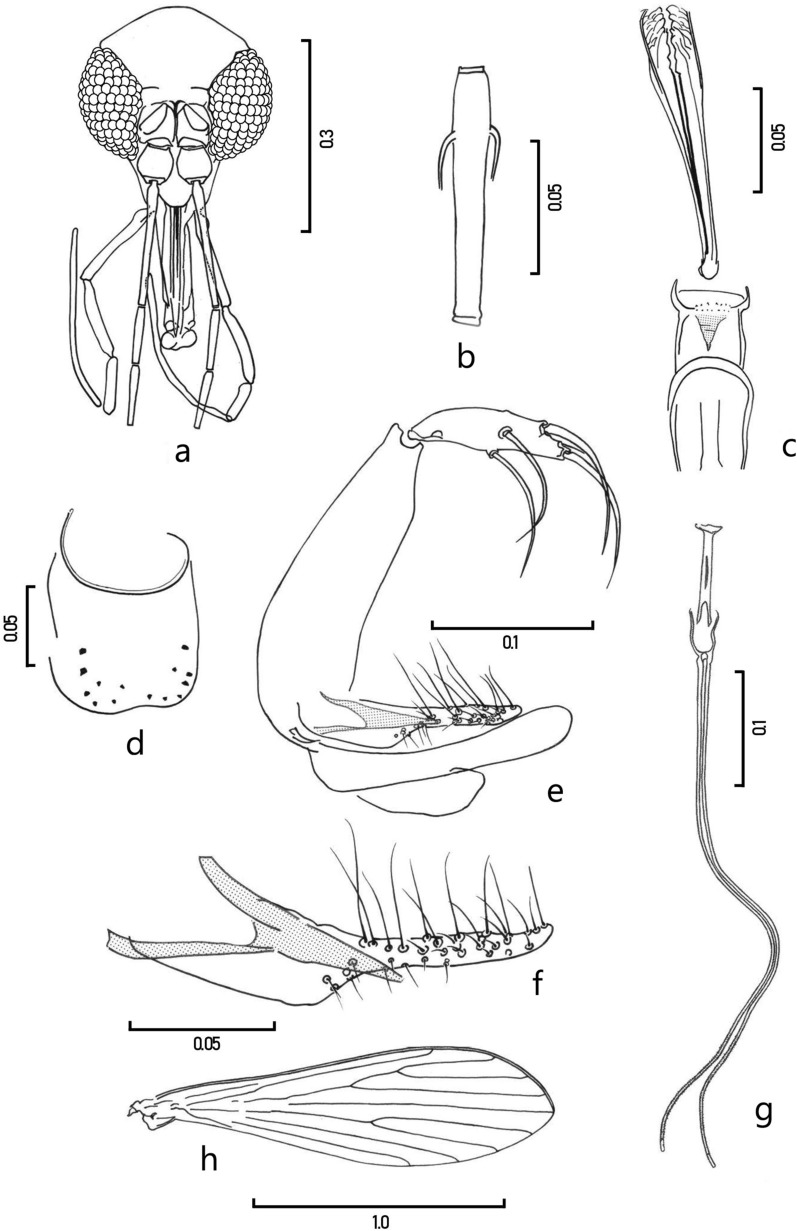
*Pintomyia* (*Pif.*) *veintemillasi* n. sp. male. **a** Head frontal view; **b** antennomer fII; **c** cibarium and pharynx; **d** sternite 2; **e** genitalia profile; **f** paramere and aedeagus, in lateral view; **g** genital pump and genital filaments; **h** wing. Scales are in mm

**Fig. 2:**
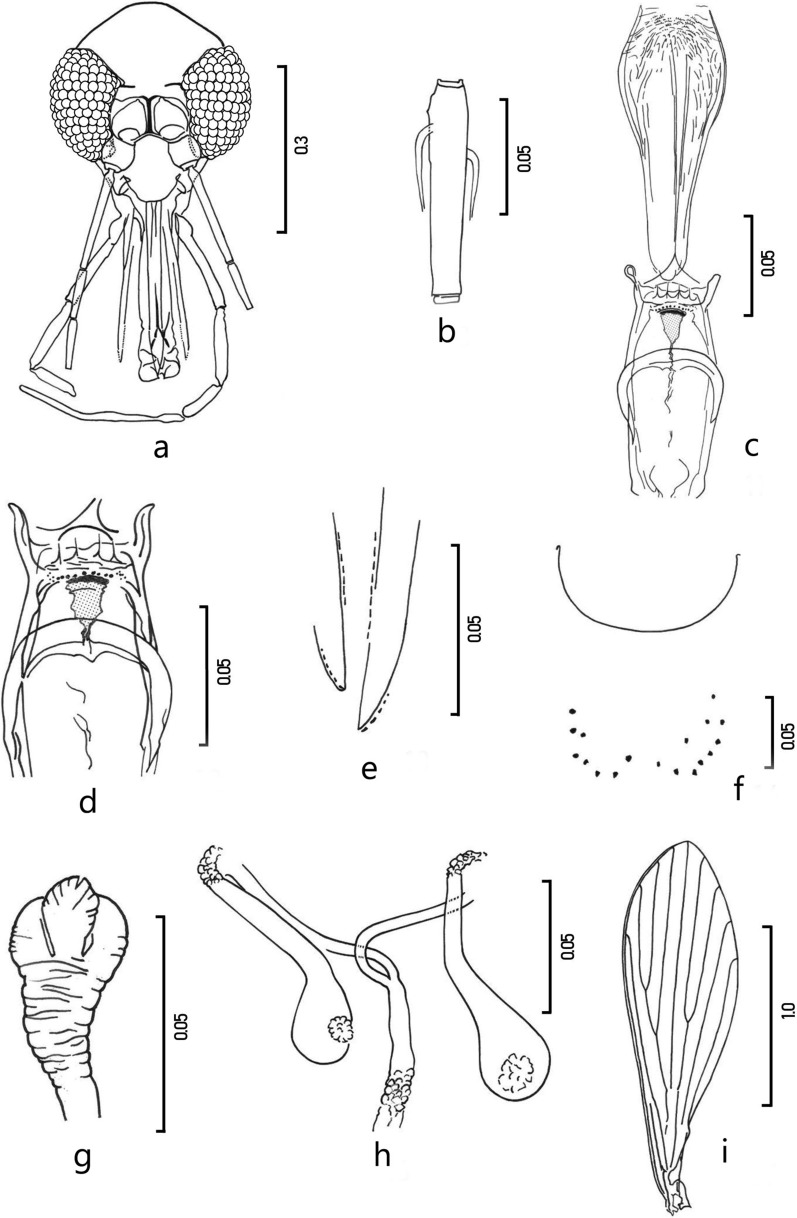
*Pintomyia* (*Pif.*) *veintemillasi* n. sp. female. **a** head frontal view; **b** antennomer fII; **c** cibarium and pharynx; **d** cibarium; **e** laciniae of the maxillae; **f** sternite 2; **g** spermatheca; **h** rapid view of a complete genitalia; **i** wing. Scales are in mm

***Pintomyia* (*Pifanomyia*) *veintemillasi* n. sp. Martinez, Leon, Mihalca, Dujardin & Le Pont** (Figs. 1, 2).

***Type locality*** Marimonos station, in the Marimonos mountain range, Bolivia. Municipality of Palos Blancos (15°35′02″S–67°15′07″W), altitude 900 m, Sud Yungas province, Department of La Paz, Bolivia.

***Type-material*** The male holotype and the female allotype are deposited in the Bolivian Fauna Collection (Colección Boliviana de Fauna, CBF), La Paz, Bolivia; 10 paratypes (five males and five females) are also deposited in CBF, La Paz, Bolivia; UPAMETROP/IINSAD (Unidad de Parasitología, Medicina Tropical y Medio Ambiente; Instituto de Investigación en Salud y Desarrollo), La Paz, Bolivia; MNHN (Museum National d’Histoire Naturelle), Paris, France.

***ZooBank registration*** To comply with the regulations set out in Article 8.5 of the amended 2012 version of the International Code of Zoological Nomenclature (ICZN) [13], details of the new species have been submitted to ZooBank. The Life Science Identifier (LSID) of the article is urn:lsid:zoobank.org:pub:E2B4956C-F39D-4075-9CCD-455E8069FBB9. The LSID for the new species *Pintomyia* (*Pifanomyia*) *veintemillasi* is urn:lsid:zoobank.org:act:81EF210D-673B-435B-B92E-7F14AC1E1F88.

***Etymology*** The species name is dedicated to Dr. Felix Veintemillas for his great contribution to the research and control of infectious and parasitic diseases in Bolivia, the main Bolivian reference on leishmaniasis in the mid-twentieth century.

## Description

Male holotype (Fig. 1). Sand fly of small size, generally gray in color, mesonotum and abdominal tergites light brown, measuring 2 mm from the tip of the labrum to the end of the gonocoxite.

*Head* (Fig. 1a). Length 0.298 (0.294–0.324) including the clypeus; maximum width 0.286 (0.284–0.321). Head length/head width ratio 1.04. Interocular distance 0.101 (0.85–0.101) equal to the diameter of 5.5 facets. Labrum-epipharynx (LE) length 0.192 (0.181–0.214) from the edge of the clypeus. Antennal flagellomeres: fI 0.171 (0.160–0.201), fII + fIII = 0.077 + 0.087. Ratio fI/LE = 0.89 (*a* = 0.91). Short ascoids, only visible on the first flagellomere. Third flagellomere without sensilla in rosette in the preapical region. Palpus: total length 0.591 (0.548–0.673), respective lengths of the palpomeres: P1 0.030 (0.025–0.039); P2 0.105 (0.102–0.127); P3 0.114 (0.108–0.125); P4 0.079 (0.077–0.095); P5 0.263 (0.202–0.311); palpal formula: 1–4−(2–3)−5 or 1–4−(3–2)−5. Cibarium armed with a row of tiny, sharp, slightly sclerotized teeth of irregular size, and an anterior, discontinuous row of dot-shaped denticles. Sclerotized arch complete, well chitinized; pigmented patch, triangular, striated, extending anteriorly. Narrow pharynx, length 0.140, maximum width 0.037, with posterior denticulate scales (Fig. 1c).

*Thorax*. Length 0.429. Unpigmented paratergite. Clear pleuras, except for the basal region of the katepisternum and katepimeron, slightly pigmented. Upper anepisternal bristles: 6 + 7 (from 5 to 10 per pleura) and proepimeral bristles: 2 + 4 (2–4 per pleura). Wings (Fig. 1h): length 1.385 (1.385–1.560), maximum width 0.405 (0.376–0.440). Length/width ratio 3.41. Wing indices: *alpha* 0.305 (0.289–0.361), *beta* 0.153 (0.136–0.174), *gamma* 0.202 (0.200–0.244), *delta* 0.099 (0.079–0.151); *alpha*/*beta* ratio 1.99 (*a* = 2.06). Leg lengths of the femur, tibia, and basitarsus, respectively: front legs 0.505–0.530–0.325; middle legs 0.549–0.660–0.420 and hind legs 0.580–0.815–0.490.

*Abdomen*. Length 1.097 including the gonocoxite. Tergal papillae present from third to seventh tergite. Second sternite with 7–9 bristles on each apical region. Gonocoxite: length 0.172 (0.172–0.193), maximum width 0.055, without perennial bristles. Gonostyle length 0.104 (0.100–0.114) bearing four strong spines: an apical spine, an upper external spine inserted in the distal third, and the spines inferior and internal implanted in mid-segment; presence of a fine spiniform, subterminal bristle. Paramere (Fig. 1f): length 0.150 (0.145–0.157), measured from the dorsal margin; rectangular base, then posteriorly finger-shaped, garnished with erect bristles, curved anteriorly. Aedeagus conical, well sclerotized, with the tip reaching the finger-shaped part of the paramere (Fig. 1f). Lateral lobe without perennial bristles, similar in size to that of the gonocoxite; length 0.177 (0.161–0.188); sub-median lamella, without particularities. Genital pump (GP) length 0.111 (0.100–0.119; *a* = 0.109); genital filaments (GF) with finely striated apical third, and smooth apex, length 0.434 (0.427–0.490; *a* = 0.448), duct/pump ratio GF/GP 3.90 (3.90–4.38; *a* = 4.12).

Female allotype (Fig. 2). Sand fly identical in coloration to that of the male, measuring 2.45 mm from the tip of the labrum to the end of the cerci.

*Head* (Fig. 2a). Length including clypeus 0.363 (0.337–0.363), maximum width 0.347 (0.325–0.348). Head length/head width ratio 1.04. Interocular distance 0.129 (0.108–0.129), equal to the diameter of six facets. Labrum-epipharynx length 0.296 (0.275–0.296) from edge of clypeus; maxillary laciniae (Fig. 2e): six external teeth and 23 internal. Antennas: length of flagellomeres, fI 0.178 (0.166–0.183), fII + fIII = 0.083 + 0.084; ratio fI / LE = 0.60 (*a* = 0.61). In fIII, absence of papilla in rosette in preapical region. Ascoids strong and short, well staggered, not reaching the apical third. Palpus: total length 0.771 (0.636–0.771). The palp segments measuring respectively: P1 0.039 (0.033–0.040); P2 0.149 (0.134–0.154); P3 0.146 (0.134–0.146); P4 0.102 (0.083–0.102); P5 0.335 (0.236–0.335). Palpal formula: 1–4−(2–3)−5, segments 2 and 3 subequal; Newstead’s sensilla not visible. Cibarium with four equidistant acute horizontal teeth of equal size; a row of 9–12 vertical teeth, and several lateral, dot shaped, grouped teeth (Fig. 2d). Very distinct sclerotized area, thickened anteriorly, triangular, narrowed at the level of the chitinous arch; this last, rounded and continuous from one edge of the cibarium to the other, surpassing it laterally (Fig. 2d). Pharynx (Fig. 2c): with the most posterior scales, denticulate; length 0.165, maximum width 0.070.

**Fig. 3:**
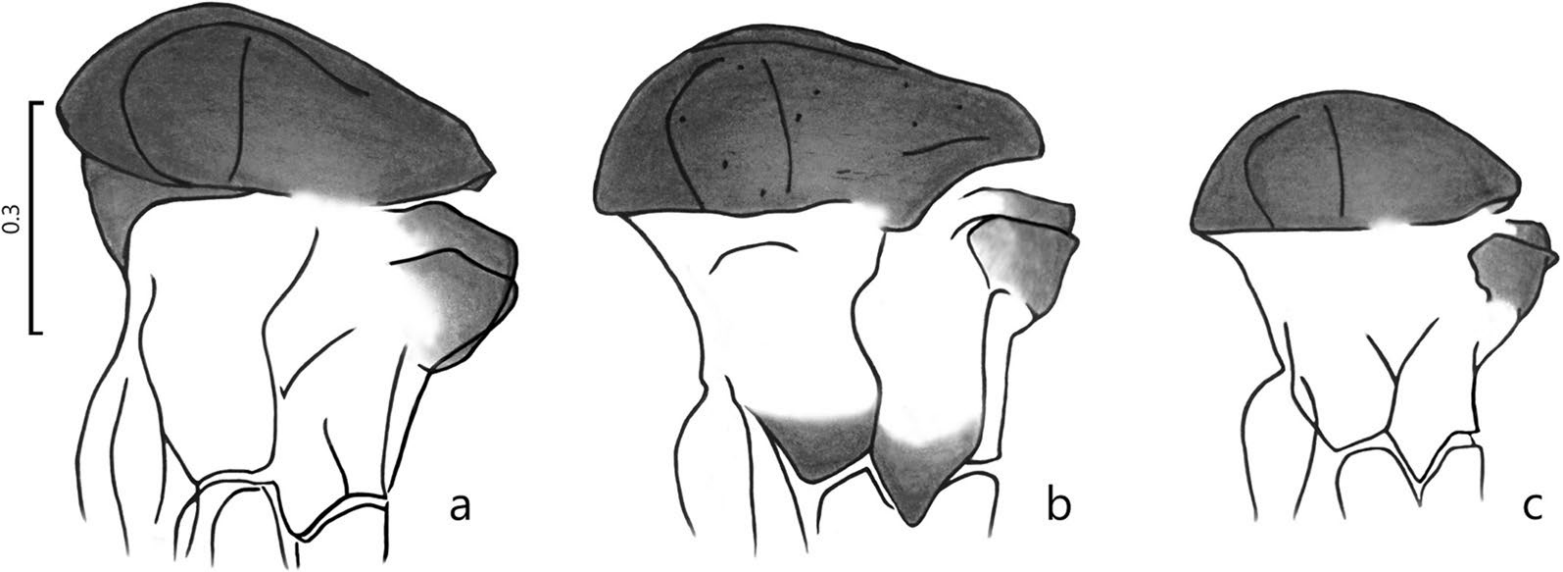
Comparative representation showing the pigmentation of the thorax profile of females (scale is in mm): **a** *Pi.* (*Pif.*) *maranonensis*; **b** *Pi.* (*Pif.*) *veintemillasi*; **c** *Pi.* (*Pif.*) *nevesi*

*Thorax* (Fig. 3b). Length 0.574. Pigmentation identical to that of the male. Upper anepisternal bristles: 7 + 11 (7–12 per pleura) and proepimeral bristles 5 + 6 (2–6 per pleura). Wings (Fig. 2i): length 1.760 (1.649–1.795), maximum width 0.525 (0.485–0.545); Length/width ratio 3.35. Wing indices: *alpha* 0.434 (0.410–0.491), *beta* 0.195 (0.187–0.224), *gamma* 0.310 (0.237–0.320), and *delta* 0.186 (0.175–0.227), *alpha/beta* ratio 2.22 (*a* = 2.16). Legs lengths of the femur, tibia, and basitarsus, respectively: front legs 0.660–0.630–0.375; middle legs 0.673–0.775–0.450 and hind legs 0.725–0.990–0.545.

*Abdomen*. Length 1.225. Second sternite with 8–10 bristles on each apical half. Spermatheca, like a pear-shaped sac, finely wrinkled transversely, head deeply invaginated in the spermatheca, with pluri-lobed apex (Table 2), fan-shaped (Fig. 2g). Importantly, the head is slightly offset from the axis of the spermatheca, and therefore most often emerges laterally after mounting (Fig. 2g). Common duct and individual ducts not measurable, but a long common duct presence.

## Discussion

Specimens of the new species *Pi.* (*Pif.*) *veintemillasi* were collected through entomological surveys carried out at both the ground and canopy levels using CDC light traps as well as protected human bait. *Pintomyia* (*Pif.*) *veintemillasi*, misidentified as *Pi.* (*Pif.*) *nevesi*, was the second most numerous anthropophilic species at the canopy level, only after *Pi*. (*Pif*.) *serrana* and the third most common anthropophilic on the ground, after *Ps. carrerai carrerai* (vector of *Leishmania braziliensis*) and *Ps. hirsutus hirsutus* (identified naturally infected by flagellates) [10, 14]. Two species of *Pifanomyia* were collected in sympatry [10, 14]; both presented similar size and a gray pigmentation: *Pi.* (*Pif.*) *serrana* (Serrana series) and *Pi.* (*Pif.*) *nuneztovari* Ortiz, 1954 (incertae sedis), syn. *Pi.* (*Pif.*) *nuneztovari anglesi* Le Pont & Desjeux 1984 [2].

Following Galati [2], the new species, *Pi.* (*Pif.*) *veintemillasi*, belongs to the subgenus *Pifanomyia*, Evansi series, according to the following morphological characteristics: palp P5 larger in length than P3 in both sexes, which is a relevant feature of the subgenus *Pifanomyia*; in the males, presence of short ascoids, reaching half of the segment, a strong four-spine gonostyle, and the presence of a preapical bristle; a gonocoxite without tuft, and a simple paramere; in the females, ascoids reaching the subapical region of the segment, a hypopharynx with deep apicolateral teeth, and a short row of external teeth for the maxillae; a cibarium with four horizontal teeth, the vertical teeth fitting into one or two transverse rows; a complete chitinous arch, and a narrow, triangular, pigmented area; spermatheca with a long common duct, vesicular body, without apical ring, transversely wrinkled, and well-individualized head. Finally, the absence of sensilla in rosette on the third flagellomer fIII in *Pi.* (*Pif.*) *veintemillasi* confirms it belongs to the Evansi series.

The morphologically similar species in this series are *Pi.* (*Pif.*) *nevesi* and *Pi.* (*Pif.*) *maranonensis* [15], noting the latter is only present on the Ecuador-Peru border. *Pintomyia* (*Pif.*) *veintemillasi* is different from *Pi.* (*Pif.*) *nevesi* based on larger size and pigmentation of the lower part of the pleura. Significantly shorter genital filaments in the new species shed light on the doubts about the criteria for identifying *Pi.* (*Pif.*) *nevesi* [6]: a genital filaments/genital pump (GF/GP) ratio = 4.12 (3.90–4.38) for *Pi.* (*Pif.*) *veintemillasi*, notably shorter than the ratio = 4.60 (4.44–4.86) established for *Pi.* (*Pif.*) *nevesi* and 4.73 (4.43–5.38) for *Pi.* (*Pif*.) *maranonensis* (Table 1). Additionally, the pigmented paratergite of (*Pif*.) *maranonensis*, in contrast to the clear paratergite of *Pi.* (*Pif*.) *nevesi*, differentiates the two species. The labrum-epipharynx of females is a good character for species distinction: *Pi*. (*Pif*.) *veintemillasi* 0.284 (0.275–0.296), *Pi*. (*Pif*.) *nevesi* 0.255 (0.233–0.266), *Pi*. (*Pif*.) *maranonensis* 0.346 (0.330–0.375) (Table 2). In the *Pi.* (*Pif.*) *veintemillasi* female (Table 2), the labrum-epipharynx is larger; the spermatheca has a purse-like shape and is finely wrinkled, with a multi-lobed head, opening out laterally, while in *Pi.* (*Pif.*) *nevesi*, it is oblong, with a fine head emerging at the apex. *Pintomyia* (*Pif.*) *veintemillasi* is only present in Bolivia in the forest regions of the sub-Andean cordillera (300–1400 m altitude) belonging to the wide basin of the high tributaries of the Amazon, whereas *Pi.* (*Pif.*) *nevesi* has been found far from this area in the lowland region.

**Table 1:** Male taxonomical characters between *Pi.* (*Pif.*) *veintemillasi* n. sp. and related species

Character	*Pi.* (*Pif.*) *maranonensis* Zumba (Ecuador) 900 m	*Pi.* (*Pif.*) *veintemillasi* n. sp. Marimonos (Bolivia) 900 m	*Pi.* (*Pif.*) *nevesi* Orthon River (Bolivia) 144 m
Pleura	Gray Pigmented paratergite Superior anepisternum and posterior anepimeron pigmented	Light color Clear paratergite Base of katepimeron and katepisternum pigmented	Light color Clear paratergite
Gonocoxite	Tuft, 2–4 bristles	Naked, without perennial bristles	Naked, without perennial bristles
GF	*a* = 0.526 (0.490–0.560) (SD ± 0.021)	*a* = 0.449 (0.427–0.490) (SD ± 0.020)	*a* = 0.520 (0.495–0.543) (SD ± 0.016)
GP	*a* = 0.111 (0.104–0.118) (SD ± 0.004)	*a* = 0.109 (0.100–0.119) (SD ± 0.006)	*a* = 0.113 (0.106–0.117) (SD ± 0.004)
GF/GP	*a* = 4.74 (4.43–5.38) (SD ± 0.30)	*a* = 4.12 (4.38–3.90) (SD ± 0.19)	*a* = 4.61 (4.44–4.86) (SD ± 0.14)

Measurements for 10 specimens of each population

(*GF* genital filaments, *GP* genital pump, *a* average, *SD* standard deviation)

**Table 2:** Female taxonomical characters between *Pi.* (*Pif.*) *veintemillasi* n. sp. and related species

Character	*Pi.* (*Pif.*) *maranonensis*	*Pi.* (*Pif.*) *veintemillasi* n. sp.	*Pi.* (*Pif.*) *nevesi*
Pleura	*Idem*, males	*Idem*, males	*Idem*, males
Labrum-epipharynx	*a* = 0.346 (0.330–0.375) (SD ± 0.015); *n* = 12	*a* = 0.284 (0.275–0.296) (SD ± 0.008); *n* = 11	*a* = 0.255 (0.233–0.266) (SD ± 0.009); *n* = 12
Spermatheca			

*a* average, *SD* standard deviation, *n* number of measured individuals. Scale 0.05

The difference is more evident with *Pi.* (*Pif.*) *maranonensis*, which has a pigmented paratergite; the male has a tuft of bristles at the gonocoxite, and the GF/GP ratio = 4.73; the female of this species has a rounded spermatheca, with a head strongly invaginated to mid-body, and emerging laterally (Tables 1, 2).

A female of *Pi.* (*Pif.*) *nevesi* was identified by Velasco and Martins (1974) from the foothills of La Paz in Huacakarita (altitude 800 m), approximately 50 km from Marimonos in the sub-Andean region [5]. Its metric data correspond to *Pi.* (*Pif.*) *veintemillasi*, confirming the presence of a cryptic species that is distinct from *Pi.* (*Pif.*) *nevesi*.

As a final remark, in Bolivia, on the La Paz-Riberalta, Beni transect, *Pi.* (*Pif.*) *veintemillasi* and *Pi.* (*Pif.*) *nevesi* presented an allopatric distribution, confined respectively to the sub-Andean (300–1400 m) and the Amazonian regions (130–200 m), separated by 400 km of the Beni plain.

In Peru, in the region of San Martin, with a geographical configuration similar to that of the department of La Paz in Bolivia, *Pi.* (*Pif.*) *veintemillasi* could represent the species, similar to *Pi.* (*Pif.*) *nevesi*, reported by Beati et al. [8]. Taxonomical studies through more recent entomological collections should give light to the possible presence of this species in Peru and neighboring countries.

This morphological description was based on mounted biological material, without available specimens for molecular studies; nevertheless, further field surveys will be conducted in the sub-Andean region to collect specimens for molecular studies needed to complete the genetic identity of this new species in comparison with similar taxa.

## Identification key for species of the subgenus *Pifanomyia*—Evansi series (updated from Galati, 2018)

**Table:** 

Males	
1	Paramere with straight dorsal margin, completely covered by spiniform setae……………. 2 Paramere with dorsal margin slightly curved at the apex towards the gonocoxite, setae restricted to this apical area ………………………………………………………… *Pi*. (*Pif*.) *ovallesi*
2(1)	Sperm pump c. 150 µm long; the pavilion’s diameter being larger than that of the sperm sac; aedeagal ducts c. 670 μm; parameres thick, the digitiform part’s width slightly smaller than that of the base and as long as the epandrial lobe ………………… *Pi*. (*Pif.*) *evansi* Sperm pump c. 120 µm long; the pavilion’s diameter being smaller or equal to that of the sperm sac; aedeagal ducts c. ≤ 570 μm; parameres thinner, with the digitiform part’s width perceptibly narrower than its base and smaller than the epandrial lobe …………………… 3
3(2)	Gonocoxite with tuft of two or more setae; thorax with paratergite brown ……………………. …….…………………………………………………………………..*Pi*. (*Pif*.) *maranonensis* Gonocoxite without tuft of setae; thorax with paratergite straw…………………….………4
4(3)	Pleura straw, relation aedeagal ducts /genital pump = 4.60 ………………… *Pi*. (*Pif*.) *nevesi* Pleura partially pigmented, relation aedeagal ducts /genital pump = 4,12 ………………… .………………………………………………………………………*Pi.* (*Pif.*) *veintemillasi*
Females	
1	Spermatheca without apical ring ………………………………………………………………………….. 2 Spermatheca with apical ring ……………………………………………………………………………… 4
2(1)	Labrum-epipharynx ≥ 330 μm; thorax with paratergite and mesonotum brown, contrasting with the straw pleura …………………………………………………………….. *Pi*. (*Pif*.) *maranonensis* Labrum-epipharynx < 300 μm; thorax with paratergite straw ……………………………………………3
3(2)	Labrum-epipharynx 275–296 μm; thorax with paratergite straw; pleura partially straw with brown pigmentation at the base (katepisternum and katepimeron)*………………………. ………………………………………………………………………………….. Pi.* (*Pif.*) *veintemillasi* Labrum-epipharynx ≤ 270 μm; Thorax with paratergite and pleura straw……. *Pi*. (*Pif*.) *nevesi*
4(1)	Spermatheca with a constriction in its apical half ………………………………. *Pi*. (*Pif*.) *ovallesi* Spermatheca without constriction in its apical half ………………………… *Pi*. (*Pif*.) *evansi*

## Conclusions

*Pintomyia* (*Pif.*) *veintemillasi* is described as a new sub-Andean sand fly that was for decades misidentified as *Pi.* (*Pif.*) *nevesi* from the Amazon lowlands, due to its similar morphology. The high anthropophily of this species could eventually be related to a vector role.

## Data Availability

The data that support the description are available from the corresponding author.
